# Current Role of Artificial Intelligence in the Management of Gastric Cancer

**DOI:** 10.3390/biomedicines13122939

**Published:** 2025-11-29

**Authors:** Efstathia Liatsou, Tatiana S. Driva, Chrysovalantis Vergadis, Stratigoula Sakellariou, Panagis Lykoudis, Konstantinos G. Apostolou, Dimitrios Tsapralis, Dimitrios Schizas

**Affiliations:** 1 Department of Surgery, Sahlgrenska University Hospital, 41345 Gothenburg, Sweden; 2 First Department of Pathology, General Hospital of Athens “Laiko”, Medical School, National and Kapodistrian University of Athens, 11527 Athens, Greece; tatianadriva@gmail.com (T.S.D.); sakellarioustrat@yahoo.gr (S.S.); 3 Third Department of Radiology, Laikon General Hospital, National and Kapodistrian University of Athens, 11527 Athens, Greece; valvergadis@yahoo.gr; 4 Fourth Department of Surgery, Attikon University Hospital, National and Kapodistrian University of Athens, 11527 Athens, Greece; p.lykoudis@gmail.com; 5 First Department of Surgery, Laikon General Hospital, National and Kapodistrian University of Athens, 11527 Athens, Greece; konstantinos.apostolou@gmail.com (K.G.A.); schizasad@gmail.com (D.S.); 6 Department of Surgery, General Hospital of Ierapetra, 72200 Ierapetra, Greece; tsapralisd@yahoo.gr

**Keywords:** artificial intelligence, deep learning, gastric cancer

## Abstract

**Background/Objectives:** In the era of precision medicine in gastric cancer, artificial intelligence has emerged as a tool in diagnosis, prognostic stratification, and clinical management. The role of big data analysis leveraging complex databases has been rapidly developing, thus challenging physicians to interpret and apply them in clinical practice. The aim of this comprehensive review is to present the current trends in the use of artificial intelligence in the field of diagnosis, histopathological evaluation, and clinical prognosis of gastric tumors. **Methods**: We screened the PubMed and MEDLINE databases for the latest studies with the development and evaluation of artificial intelligence algorithms in gastric cancer. Different sorts of deep learning protocols were explored, and their standardized applications are presented herein. **Results**: A broad spectrum of AI-based models extending from the surveillance of high-risk subepithelial lesions to the precise molecular characterization of tumors and treatment management are gaining space in clinical practice. However, all current studies are lacking a randomized design at the large-population scale, which is required to further integrate machine learning algorithms into standard clinical care. **Conclusions**: Despite the remaining challenges of data quality, algorithm improvement, and outcome interpretation, there is promising evidence that artificial intelligence can revolutionize gastric cancer management. There is a need to develop AI algorithms based on big data sources that must consequently be evaluated in randomized multicenter studies.

## 1. Introduction

Gastric cancer remains a leading cause of cancer-related mortality worldwide, with early diagnosis and accurate staging representing pivotal factors in improving clinical outcomes [1]. The prognosis of advanced gastric cancer is poor, with a 5-year-survival rate of 30%, while the same rate for early gastric cancer (EGC) can reach 90% [2,3,4].

Endoscopy is the most efficient method for the diagnosis of EGC lesions, with its sensitivity depending on factors such as the location of the lesion, the endoscopist’s skill, lesion morphology, and the number of biopsies taken [5,6]. However, upper endoscopy still lacks optimal sensitivity for EGC detection, as it has been shown that up to 10% of cancers are missed even after the procedure is performed [7]. Upon the realization of advanced disease states, systematic treatment based on chemotherapy regimens, radiotherapy, and target therapy can prolong survival to some extent, with significant challenges and clinical dilemmas [6]. As the overall survival of patients with gastric cancer increases, precision medicine and personalized prognostic stratification are gaining emphasis and have become more complicated, [8] leading to the design of prognostic models including multiple time-varying variables which are able to estimate long-term outcomes following surgery [9,10].

The emergence of big data has transformed the landscape of modern medicine. For physicians, the challenge has shifted from data collection to the effective interpretation and analysis of vast and complex datasets [11].

The concept of artificial intelligence (AI) as the capability of automated systems to learn and exhibit intelligence plays a pivotal role in this transformation. With the advent of precision medicine, AI facilitates the conversion of big data into clinically meaningful insights, helping to reduce inevitable human errors, enhance diagnostic validity, enable dynamic analysis in real time, and even support postoperative care [12,13,14]. As a result, AI is fundamentally revolutionizing and reshaping the approach to gastric cancer management [15,16].

The aim of this review is to explore the published literature and present the applications of AI in the diagnosis, treatment, and prognosis of gastric cancer, while examining how deep learning and convolutional neural networks (CNNs) have evolved in the medical big data field during the past decade.

## 2. Data Collection Procedure

**Figure 1:**
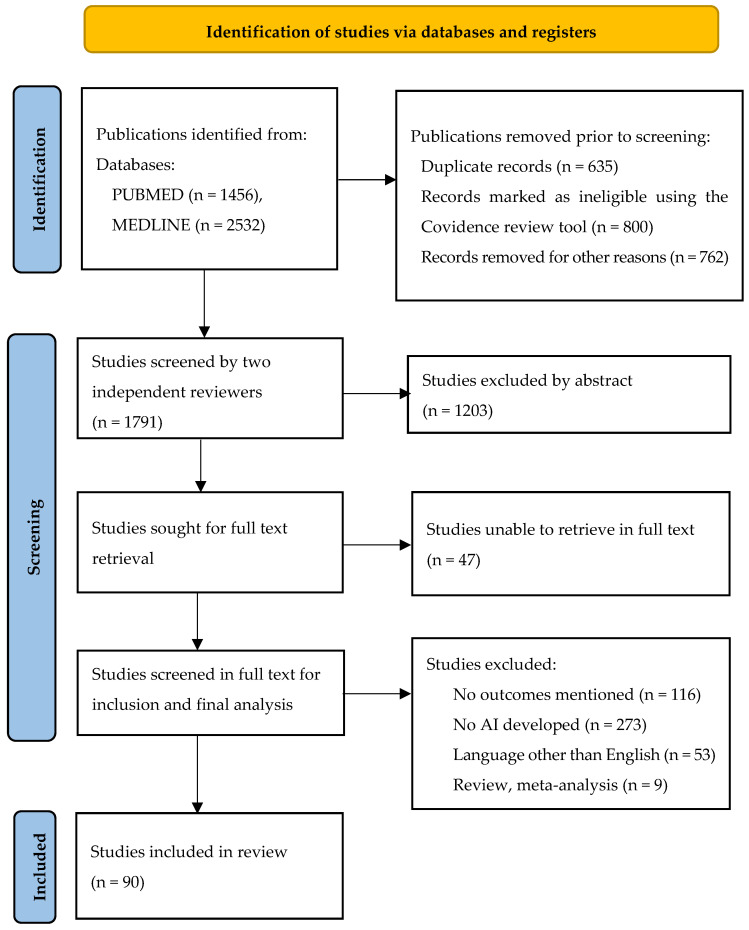
PRISMA 2020 study flow chart with the screening process and inclusion of studies.

The PubMed and MEDLINE databases were scanned from their inception until April 2025. The main search algorithm was the same for the aforementioned databases and included the following terms: (Artificial Intelligence OR Machine Learning OR Learning Algorithms OR Deep Learning OR Convolutional Neural Networks OR Unsupervised Machine Learning OR Supervised Machine Learning) AND (Gastric cancer OR Gastric tumor OR Gastric Adenocarcinoma OR Stomach Neoplasms OR Gastric Malignancy) [Supplementary Concept] [Mesh]. The PRISMA flow diagram demonstrates the article selection process (Figure 1). Inclusion criteria involved prospective and retrospective cohort studies and randomized clinical trials relevant to the development of AI techniques for the diagnosis, imaging, histopathology assessment, prognosis, and surgical management of gastric cancer. Studies should present metrics of performance evaluation and be published in English. Studies with traditional statistical models, addressing other malignancy types, studies with no outcomes, and studies published in a language other than English were excluded. Relevant articles were also found by scanning the references of identified articles (backward search) and locating newer articles that included the original cited papers (forward search). Study selection was performed in three consecutive stages by two independent reviewers. First, duplicate publications were removed, and then the titles and abstracts of all electronic articles that appeared in the search were read in order to assess their eligibility. The Covidence review tool was used for screening of studies. Second, full texts of all articles that met the inclusion criteria were downloaded, and all observational studies were selected. Study search and data tabulation were conducted by two authors on a predefined form. A third author resolved any possible conflicts after retrieving all available data. The structure and presentation of the review followed the PRISMA checklist 2020.

## 3. Results

### 3.1. AI-Assisted Gastric Cancer Screening and Diagnosis with Endoscopy

**Figure 2:**
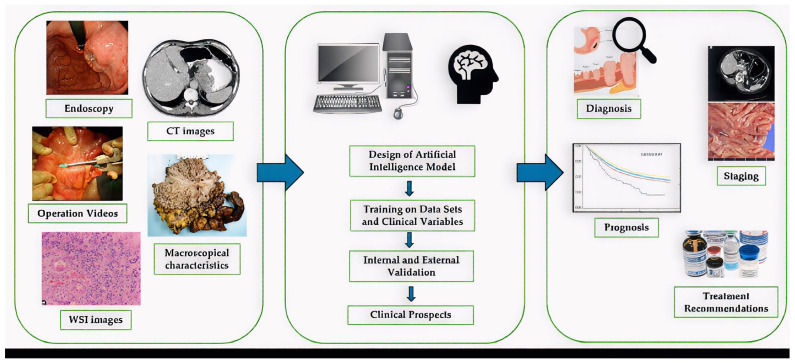
Working process for the development and implementation of artificial intelligence in the clinical care of patients with gastric cancer.

The basis of application of artificial intelligence in the field of surgical oncology is based on the field of radiomics in radiology, the special field of medical imaging that involves the acquisition of extensive quantitative features from medical images, which are not visible to the naked eye but can be analyzed by algorithms to provide valuable clinical information. By applying complex algorithms, radiomic features can be combined and co-analyzed with demographic, histologic, genomic, and proteomic features to answer clinical questions as shown in Figure 2. In the field of AI development, gastric cancer screening has emerged as a major research focus, encompassing related tasks such as the diagnosis of *Helicobacter pylori* infection, the detection of atrophic gastritis, the identification of EGC, and the prediction of tumor invasion depth [16,17].

In this framework, high accuracy AI-based imaging and analysis methods have been developed to assist the diagnostic capabilities of endoscopy as summarized in Table 1. A specialized approach in image analysis using convolutional neutral networks (CNNs) enables the elaboration and extraction of visual features from a source image through nonlinear activation functions, followed by processing with pooling and dimensionality reduction [18]. Different deep learning algorithms, such as SVM (Support Vector Machine), Inception ResNEt, and SSD (Single Shot Multibox Detector), have been used in combination with the CNNs to classify feature values from different diagnostic endoscopic methods [19]. Ikenoyama et al. compared the diagnostic performance of a CNN with that of endoscopists [20]. The CNN was trained on 13,584 esophagoduodenoscopy images from 2639 biopsy-confirmed gastric cancer lesions collected from four medical institutions in Japan [20]. The performance of 67 endoscopists was then compared with that of the CNN on the same images. The CNN showed a significantly higher sensitivity, specificity, and positive predictive value (PPV), with a comparable ability to rule out gastric cancer (NPV) [20].

However, while the CNN achieved higher diagnostic sensitivity even among experienced, certified endoscopists, false positive results remained high especially in cases with non-neoplastic conditions such as chronic gastritis and intestinal metaplasia. The CCN maintained consistently higher sensitivity compared with non-certified endoscopists, highlighting its potential to assist novice endoscopists with daily diagnostic routines as well as reduce the physicians’ workload and spare resources for healthcare systems in poor countries. With respect to false negative results, the CNN most often missed small-sized lesions with diameters less than 10 mm, whereas gastritis was found to be the most frequent cause of false negatives among endoscopists [20].

Estimation of invasion depth remains a key factor in determining treatment options for EGC. Endoscopic ultrasonography (EUS) facilitates this evaluation, as mucosal or minimal submucosal invasion allows for endoscopic submucosal dissection of the tumor [21]. Τhe diagnostic reliability of EUS for T staging of EGC varies widely, ranging from 70 to 90%, a phenomenon attributed to the individual diagnostic skills of the practicing physician [22,23].

A computer-aided diagnostic system for EUS was trained using a dataset of 420 consecutive cases of newly diagnosed EGC from Osaka University, collected between June 2009 and December 2019. This dataset generated 3451 EUS images, which were used to train both segmentation and classification AI models. The models were then prospectively validated on internal and external datasets (1726 and 3103 study EUS images, respectively) [24]. In internal validation, the model outperformed endoscopists with significantly high accuracy; however, this was not consistent with image datasets from other institutions [24]. Differences in image acquisition equipment during endoscopy affected the system’s performance, thus stressing the need for image calibration systems such as Generative Adversarial Networks (GANs) (“domain shift” problem) [25,26].

The AI model did not show any specific improvements in diagnostic accuracy on particular histological types as well as when combined with contrast-enhanced EUS (CE-EUS) and expert interpretation [24]. This fact ensures the models’ ability to maintain their high diagnostic performance despite limited human and technological resources. One limitation of this study relates to the restricted number of images ultimately included, as many were excluded due to poor quality.

In routine clinical practice, endoscopists often make diagnoses based on images of limited quality, supplemented by patients’ medical histories and their presenting symptoms. In contrast, AI models lack this flexibility, resulting in a high proportion of non-evaluable images. Segmentation of the lesions in regions of interest (ROIs) remains from the endoscopic images which enables advanced detection and classification with high sensitivity as demonstrated in heterogenic study methodologies with different datasets, algorithms, and metrics [27,28].

However, existing retrospective studies provide a training dataset with still images, which do not reflect the variability of real-time clinical practice, including changes in angles and imaging artifacts [29]. This was depicted in the first-ever published prospective study by Kim et al., who attempted to train an AI model using real-time endoscopic video footage to assess invasion depth in EGC [1]. The AI model that was previously trained in static images lacked significant diagnostic accuracy and reliability for tumor invasions’ depth when compared with endoscopists in the real-world scenario [30]. This has raised the need for the development of a video classifier model that is able to process sequential video frames through convolutional layers, which was used, and diagnostic performance significantly improved compared to image-trained systems [30].

**Table 1:** Included and analyzed studies developing and assessing the use of AI for gastric cancer screening and early diagnosis.

Author, Year	Dataset	Data Size	AI Model Used	Outcome
Ikenoyama et al., 2021 [20]	Stomach images for gastric cancer lesions	Training dataset: 13,584 images for 2369 histologically proven gastric cancer lesions Validation dataset: 69 consecutive patients, 77 gastric lesions	Single Shot Multibox Detector	Sensitivity/Positive Prognostic Value for identifying early gastric cancer.
Uema et al., 2024 [24]	Endoscopic ultrasonography images from patients with early gastric cancer	Training dataset: 3889 EUS images from 285 EGCs Validation dataset: 1726 EUS from 13mi5 EGCs (internal) −3346 EUS images from 139 EGCs (external)	PyTorch/ ResNet34	Classification of lesions as mucosal cancer, submucosal cancer, or invasive lesions.
Kim et al., 2022 [30]	Endoscopic images and video clips of either mucosal or submucosal cancer	Training dataset: 1582 static images of mucosal and 1697 of submucosal cancer/189 and 165 video clips of submucosal cancer, respectively Validation dataset: 84 video clips of mucosal and 46 of submucosal cancer	VGG-16 image classifier and video classifier (IC and VC)	Prediction of invasion depth of gastric cancer lesions.

### 3.2. Clinical Staging

Accurate tumor staging of gastric cancer, as defined by the American Joint Committee on Cancer (AJCC), is crucial for the appropriate treatment of gastric cancer, especially in patients with locoregional disease who may benefit from curative surgery [31]. Imaging approaches each have strengths and limitations, especially in lymph node involvement assessment. Computed tomography (CT) is routinely applied for preoperative node (N) staging and detection of metastases [32], but despite technological advancements, its diagnostic accuracy remains limited to 50–70%, primarily due to subjective human interpretation, highlighting the need for deep learning-based radiomic algorithms [33].

In a multicenter study, Dong et al. enrolled 730 patients from four medical centers in China with locally advanced gastric cancer (LAGC) to develop a deep learning radiomic nomogram (DLRN) based on the images from multiphase CT images obtained 2 weeks before surgery [34]. The N stage was confirmed by pathology reports [34]. In the validation set from the same center, the DLRN achieved strong discriminatory performance for N staging, outperforming clinical N stage criteria (short-axis diameter and radiological characteristics) and reproduced high accordance with the real N stage in different patient subgroups [34]. The whole reporting process takes 5 min per patient which makes the system easy to use in every day clinical practice. The main strength of the study was that DLRN detected 81,7% of the non-typical lymph node metastasis, suggesting it can function as a complementary tool in the preoperative staging workflow when CT findings are ambiguous.

However, the use of an Italian external validation cohort raised concerns regarding the generalizability of AI models trained on databases with differing biological and histopathological characteristics [34]. To address this challenge, the researchers developed the National Biomedical Imaging Archive (NBIA) as a standardized open-source database available for AI training to enable prospective research on gastric cancer TNM staging [35].

More advanced versions of lymph node metastasis (LNM) evaluation using CT imaging have been developed by Jin et al. Their ResNet-18 CNN composed of 11 isomorphic subnetworks was trained on 1172 patients and externally validated on 527 patients [36]. The model demonstrated powerful predictive power across all lymph-node stations, with enhanced visualization ability in the case of intratumor heterogeneity [36]. Interestingly, combining clinical factors did not significantly improve prediction, suggesting that LNM risk prediction largely depends on imaging features of the primary tumor, a fact that highlights the need for an AI system to securely and repeatedly determine the lymph node status [36].

In metastatic disease, peritoneal metastasis (PM) represents the most frequent presentation and a key prognostic indicator. However, 10-30% of patients with PM show no detectable findings on a CT scan [37]. Radiomics-based approaches have been developed and evaluated in clinical studies aiming to optimize the detection accuracy [38]. Until now the main emphasis has been the peritoneal region when identifying PM. The study by Jin et al. revealed that imaging characteristics of both the primary tumor and peritoneum are related to occult PM, offering radiological backing for the “seed-and-soil” theory [36]. The last finding supports that the radiomics of “potential regions of interest” for metastasis (soil) could be calculated by AI models, instead of focusing on the radiological characteristics of the primary tumor “seed”. Similarly, Huang et al. applied a CNN model to predict the occult PM in 544 patients, outperforming the clinical model [10]. A new era of metastasis prediction has been formulated by Jiang et al. who proposed the Peritoneal Metastasis Network (PMetNet), a densely connected CNN with long–short connections designed to predict occult PM based on preoperative CT images [39].

### 3.3. Histopathology and Molecular Classification

Gastric cancer represents a heterogeneous histological entity in which histopathological diagnosis with hematoxylin and eosin (H&E)-stained slides faces several challenges [40]. As precision medicine and targeted therapies advance, demand is increasing for AI systems capable of delivering accurate reproducible histopathological diagnoses. The current literature on deep learning (DL) in whole-slide imaging (WSI) has focused on three applications: (1) primary histological diagnosis and characterization of tumor components with prognostic relevance; (2) assessment of clinically important immunohistochemical staining; and (3) molecular analysis for biomarker detection and prognostication [41]. Studies with available data on the development and use of artificial intelligence algorithms in staging and histopathological and molecular profiling of gastric cancer are presented in Table 2.

Historically, the rationale for applying DL in gastric cancer diagnostics was conceived and presented by Karakitsos et al. who performed a series of studies using gastric smears stained with the Papanicolaou technique. Their custom image-analysis system was trained on 2500 cells from patients diagnosed, while 8524 cells were used as a test set (11,024 cells in total from both benign and malignant gastric lesions) [42]. The Learning Vector Quantization (LVQ)-based artificial network achieved superior accuracy in comparison with cytologists, particularly in cases with subtle cytological atypia [42]. However, the lack of independent external validation limits the generalizability of the results. The “e-Pathologist” was trained on H&E slide images to categorize colorectal lesions as carcinoma, adenoma, and non-malignant [43]. The high sensitivity with small underestimation rated results suggested the potential of an automated histology evaluation system to reduce the burden of work and double checking [43].

All of the following studies share the same limitation of conventional FFPE slide techniques which require processing time and involve production artifacts [44,45,46,47]. To address this, Cho et al. integrated AI-based real-time histopathology into the confocal laser endomicroscopy system (CLES) which enables high-resolution imaging and analysis of gastric tumor biopsies [48]. A total of 43 fresh tissue samples from patients diagnosed with gastric cancer and operated on were used to train a specialized AI model processing kilopixel grayscale images. The performance of dynamically distinguishing the cancer from normal tissue followed by determination of the histological subtype was comparable to that of the pathologists, and its standalone ability emphasized its potential.

Regarding subclassification of gastric tumor, a major challenge is the automated PD-L1 combined positive score (CPS) interpretation, which is more complex in gastric cancer than the tumor proportion score (TPS), as it requires evaluating both PD-L1-positive tumor cells and mononuclear inflammatory cells in the stroma [49,50]. Badve et al. presented the first AI model for automated PD-L1 CPS scoring at ASCO 2024, analyzing 97 gastric cancer WSIs against the manual evaluation of 12 pathologists [51]. The AI model achieved higher overall concordance than the interobserver agreement between pathologists, though discrepancies emerged in patients with a high CPS score, where AI classified at least 50% more patients as positive. This shows that by using AI we can safely screen patients for PD-L1 expression with a high concordance rate with pathologists, nullifying the need for a second opinion [51].

The tumor microenvironment (TME), characterized mainly by the distribution of tumor-infiltrating lymphocytes (TILs) in peritumoral regions, correlates with PD-L1 expression by the tumor and immune cells and therefore predicts the response to immunotherapy [52,53]. The classification of gastric tumors into immune phenotypes using AI-based algorithms has not yet been established in clinical research [54,55]. Li et al. presented the only study with robust data on the enumeration and quantitative assessment of TILs on gastric adenocarcinomas applying a ResNet-18 model to classify WSIs into three tertiary lymphoid structure (TLS) grades [56]. Tertiary lymphoid structures are ectopic lymphoid organs that develop in non-lymphoid tissue, and their function has been shown to demonstrate anti-tumor modalities. This study proposed an automated and repeatable way to characterize and quantify those regions that can also be used as a complementary tool in prognosis prediction [56].

More refined histopathology assessment can be enhanced with the development of AI models for Epstein–Barr virus (EBV) status and microsatellite instability (MSI), both of which are strong indicators of immunotherapy response (e.g., pembrolizumab). Muti et al. [57] validated a deep learning (DL) model across 2823 patients from ten international cohorts, demonstrating variable performance influenced by patient sex, disease stage, and histological subtype. Similarly, Kather et al. trained an AI model using TCGA data to identify MSI, with successful external validation in an independent Japanese gastric cancer cohort and enhanced accuracy when combined with pathologists [58]. Wang et al. further enhanced this approach by developing an ensemble DL model using over 370,000 image tiles from TCGA whole-slide images, supporting the robustness and generalizability of AI-based molecular prediction in gastric adenocarcinoma [59]. The biopsy review time was also reduced as the DL system could automatically integrate with the pathologists’ review.

HER2 status, another critical biomarker, traditionally requires labor-intensive and costly histopathological evaluation [60,61]. Novel DL algorithms are being developed to allow HER2 status assessment from H&E-stained gastric tumor WSIs, a variable which is rarely assessed by pathologists due to high cost and long process time [62]. Sharma et al. proposed a nuclei-based relational graph method to classify cells as non-tumor, HER2-positive, and HER2-negative [63], outperforming the widely used “multiple texture, color and intensity feature” [63]. More recently, a pixel-level tumor detector called HER2Net was introduced by Liao et al. that outperformed the classical deep learning systems [62]. Several limitations regarding misclassification due to small sample size need to be addressed. The AI model was more vulnerable to poorly differentiated ducts with high nucleocytoplasmic ratios, and stromal fibrosis while linear or clustered nuclear patterns with scant cytoplasm and hyperchromasia affected the specificity of the algorithm [62].

**Table 2:** Summary of studies with artificial intelligence algorithms for staging and histopathological and molecular profiling of gastric cancer.

Author, Year	Dataset	Data Size	Method Used	Outcome
Dong et al., 2020 [34]	Unenhanced and contrast-enhanced CT images of patients with locally advanced gastric cancer	Training set: CT images from 225 patients with locally advanced gastric cancer Validation set: 505 from different	DenseNet-201 (Deep Convolutional Neural Network)	Discriminative ability of the deep learning algorithm when assessing lymph node stage and overall survival
Jin et al., 2021 [36]	CT scan images of patients with gastric adenocarcinoma before partial or total gastrectomy	Training set: 1172 patients Validation set: 527 patients	Unet with ResNet-108	Prediction performance of lymph node metastasis
Huang et al., 2021 [10]	Histopathology pictures of patients with gastric cancer	Training set: 2333 H&E pathological pictures of 1037 cases Validation set: 179 digital pictures of 91 cases	GastroMIL and MIL-GC	Predictive performance in differentiating gastric cancer from normal tissue in H&E biopsies.
Jiang et al., 2021 [39]	CT images of patients with histopathologically confirmed gastric cancer	Training set: 1225 patients who underwent gastrectomy Validation set: 504 patients (cohort 1); 249 (cohort 2)	Peritoneal Metastasis Network (PemNet)	Ability to recognize peritoneal metastasis
Karakitsos et al., 1998 [42]	Gastric smears stained by the Papanicolaou technic	Training set: 2500 cells of 23 of patient with cancer, 19 with gastritis, and 58 with ulcus Validation set: 8524 cells of respective cases	Learning Vector Quantization (LVQ)	Discrimination between benign and malignant gastric lesions
Cho et al., 2024 [48]	Images of gastric adenocarcinoma and normal tissue samples acquired with confocal laser endomicroscopic system (CLES)	Validation set: 3686 (internal) and 100 CLES images (external)	Convolutional Neural Network	Discrimination between benign and malignant gastric lesions
Li et al., 2023 [56]	Hematoxylin–eosin-stained images of patients with gastric cancer	353 patients with gastric cancer	ResNet-18	Tumor detection and classification of Tertiary Lymphoid Structures
Badve et al., 2024 [51]	Whole slide images (WSIs)	97 biopsies with gastric cancer stained for PD-L1	Mindpeak Gastric PD-L1	Pairwise concordance between histopathologists and AI software for CPS score
Muti et al., 2021 [57]	Digitized histological slides from FFPE gastric cancer with matched microsatellite instability	2823 patients with known microsatellite instability and 2685 with known EBV status (10 centers in 7 countries)	ResNet-18	Deep learning detection of EBV in gastric cancer biopsies
Kather et al.,2019 [58]	FFPE samples of gastric adenocarcinoma	Training set: 360 patients; 93,408 tiles External validation set: 378 patients; 896,530 tiles	ResNet-18	Ability to detect MSI status in H&Ε gastric cancer biopsies
Wang et al., 2022 [59]	H&Ε samples of gastric adenocarcinoma	332 microscopic images of gastric cancer biopsies	EfficientNet-b1	Infer molecular subtypes of gastric cancer
Sharma et al., 2016 [63]	WSIs with HER2 and H&E stain, acquired from surgical sections of distinct patients of gastric adenocarcinoma	Training set: 11 WSI Validation set: 795 tiles	Random forests machine learning	Suitability of cell nuclei attributed graph (cell nuclei ARG) to molecularly classify gastric tumors
Liao et al., 2025 [62]	H&E tiles of the Internal-Stomach Adenocarcinoma dataset (STAD)	Training set: 531 H&E WSIs from 63 HER-2 positive patients and 457 HER-2 negative patients Validation set: 115 H&E WSIs from 17 HER-positive and 94 HER-2 negative patients	Pixel-Level Tumor Detector	Prediction of HER2 status in H&E gastric cancer biopsies

### 3.4. Prediction of the Clinical Outcome and Prognosis and Treatment Suggestions

Prognostic tools are mainly based on conventional Cox proportional hazard (CPH) models, which calculate outcomes using weighted prognostic factors as summarized in Table 3 [64,65]. These models have already been successfully applied in colorectal, hepatocellular, and breast cancer and sarcomas [66,67]. In gastric cancer, Huang et al. developed the prognostic GastroMIL model, which identified and extracted the most suspicious tiles from 199 malignant pathological slides from Renmin Hospital of Wuhan University (RHWU) and from 440 slides from the TCGA cohort, based on histopathological features such us necrosis, nerve invasion, signet ring cells, intravasated cancer cells, muscularis propria invasion, and mucous secretion [10]. The model then input values ranging between 0 and 1 into a prognostic framework to classify patients into high- and low-risk subgroups [10]. The DL model outperformed human pathologists, and its main strength lied in its ability to elaborate suspicious tiles [10]. The authors implemented GastroMIL through an online website making it easy to access and be used by clinicians without experience in AI formats.

Following this, four additional studies implemented artificial neural network (ANNs) in prognostic modeling for gastric cancer. Que et al. developed a preoperative ANN for predicting 3-year overall survival, achieving high accuracy rates [68]. Afrash et al., Oh et al., and Li et al. predicted 5-year overall survival, with reported AUC values of 0.935, 0.81, and 0.84, respectively [69,70,71]. A common limitation across these studies was that the proposed AI models primarily incorporated only tumor diameter and TNM stage, which reduced their generalizability and ability to evaluate complex factors such as age, sex, inflammatory response, and nutritional status. More complex DL algorithms, able to combine multiple laboratory and clinical variables to predict overall survival, have recently emerged [72]. Kuwayama et al. compared four different DL techniques using data from 1678 patients who were operated on for gastric cancer at Chiba Cancer Center [54].

The concordance between treatment guidelines proposed by a multidisciplinary tumor board (MTB) and those suggested by AI was evaluated in a retrospective study of 322 consecutive gastric cancer patients at a university hospital in Korea [73]. Concordance was assessed in two categories: (a) “recommended” and (b) “for consideration”. Overall, concordance rates between AI and MTB were 72.98% at the recommended level and 89.96% at the consideration level [73]. The lowest level of concordance was observed in Stage IV patients (45.83%) and in older patients with clinical comorbidities and poor compliance with chemotherapy [73]. Notable differences in treatment recommendations between AI and MTB were observed across cancer stages [73]. This discrepancy was mainly attributed to the different local guidelines embedded in each S-1, plus cisplatin is a well-established and widely used scheme in Korea and Japan thus recommended by MTB, while National Comprehensive Cancer Network (NCCN) guidelines (underpinning the AI’s Watson for Oncology platform) classify S-1 as experimental and do not include it.

These findings highlight the need to establish standardized AI-assisted programs aligned with regional guidelines, which vary across races, countries, and healthcare systems [73]. Moreover, cautious interpretation of AI recommendations is necessary, as they often prioritize cost and time effectiveness of regimens, whereas physicians’ decisions tend to be driven by survival outcomes [74].

### 3.5. Surgical Procedures

In recent years, substantial advancements have been observed in the integration of artificial intelligence into surgical practice, particularly concerning the intraoperative and postoperative management of gastric cancer procedures [75]. Laparoscopic gastrectomy, however, continues to be a technically demanding and prolonged intervention [76]. Consequently, the systematic video documentation of surgical instrumentation and operative protocols has emerged as a critical component for both the external validation of surgical techniques as well as the advancement of education and training within the surgical community [77,78].

Yamazaki et al. developed an automated system for detecting surgical instruments in laparoscopic gastrectomy videos, leveraging the open-source convolutional neural network platform YOLOv3 [79]. The system was trained on 10,716 images extracted from 52 laparoscopic gastrectomy videos over 200,000 iterations from the gastrectomies performed at the Surgery Department of University Hospital of Kobe [79]. The system achieved high precision in detecting surgical devices with heat maps [79]. Three experienced surgeons evaluated these heat maps and accurately identified key procedural details, such as the type of anastomosis, the time to initiate duodenal and gastric dissection, and the occurrence of any unclear procedures, with correct answer rates exceeding 90% [79].

In addition, a context-aware computer-assisted surgery system (CA-CAS) has been developed to enable visualization of both preoperative and intraoperative aspects of sigmoidectomies [80]. Using a CNN, the model was trained on annotated surgical videos and demonstrated high accuracy in recognizing various stages of the surgical workflow [80]. Additionally, the model was able to perform in near real-time, processing at 10 frames per second [80].

In the intraoperative field, Sato et al. presented a preliminary study focused on the development of an AI-driven navigation system to assist in gastric cancer surgery [81]. The AI model was trained on 1242 annotated intraoperative images from six patients, focusing on delineating the pancreatic contour [81]. The system achieved a median maximum intersection over union (IoU) score of 0.708, surpassing the threshold of 0.5, indicating a substantial improvement in contour detection [81]. However the AI system encountered challenges in accurately identifying the pancreatic contour in areas obscured by fatty issue or thin vessels [81].

More recently, Takeuchi et al. aimed to correlate radical gastrectomy with lymph node dissection with surgical complexity and to establish an AI model that could generate postoperative outcomes based on perioperative factors [82]. The surgical procedure was divided into 10 phases, and the surgical complexity score was based on extended total surgical time, intraoperative bleeding, and the presence or absence of postoperative complications classified as grade 1 or higher according to the Clavien–Dindo classification [82]. The AI model, TeCNO, a multi-stage temporal convolutional network, was trained using 75% of video data derived from 56 patients who underwent robotic distal gastrectomy with D1 or D2 lymphadenectomy and was then tested on the remaining 25%. The AI model achieved an overall accuracy of 87% in recognizing surgical phases [82]. The highest sensitivity (62%) was encountered during the Billroth-I reconstruction phase, while the highest accuracy (96%) was reached during duodenal resection phases.

In the era of minimally invasive surgery with robotic techniques, Li et al. conducted a large-scale, multicenter retrospective cohort study comparing the short- and long-term outcomes of robotic gastrectomy versus laparoscopic gastrectomy for gastric cancer [83]. The overall complication rate was lower in the robotic gastrectomy group (12.6% versus 15.2%) [83]. Additionally, robotic gastrectomy demonstrated advantages such as reduced blood loss (126.8 mL versus 142.5 mL), more lymph nodes dissected (32.5 versus 30.7), and better management of the suprapancreatic region (13.3 versus 11.6) [83].

Postoperatively, research has focused on developing predictive AI models for complications following gastrectomy. In a retrospective study by Chien et al., data from 521 patients from three hospitals in Taiwan were processed, including preoperative nutritional status, tumor characteristics, and intraoperative surgical details [84]. Three different AI models were trained—artificial neural networks (ANNs), Decision Tree Analysis (DT), and Logistic Regression (LR)—and tested for their accuracy in predicting postoperative complications [84]. The ANN model achieved the highest predictive accuracy compared to DT and LR, with accuracy rates of 81.4%, 73.3%, and 75.2%, respectively [84]. The ANN was particularly effective at capturing the complex, nonlinear relationships between clinical variables and postoperative outcomes [84]. Important predictors across all models included serum albumin, tumor stage and size, extent of surgery, and intraoperative blood loss [84].

**Table 3:** Included studies on the development and assessment of AI models for prognostic outcomes and surgical procedures.

Author, Year	Dataset	Data Size	Method Used	Outcome
Huang et al., 2021 [10]	H&E gastric cancer	2333 images of 1037 patients with gastric cancer	GastroMIL	Accuracy for diagnosis of gastric cancer and prognostic outcome
Que et al., 2019 [68]	Patients with gastric cancer from tertiary hospital–clinical and laboratory values	Training set: 1104 patients Testing set: 504 patients	Artificial neural network (ANN)	Prognostic ability of ANN for gastric cancer
Kuwayama et al., 2023 [54]	Patients who underwent surgery for gastric cancer/35 clinicopathological preoperative variables	1687 patients	Logistic Regression (LR), Random Forest (RF), Gradient Boosting (GB), Deep Neural Network (DNN)	Prognostic evaluation for survival
Park et al., 2023 [73]	Patients who underwent surgery for gastric cancer	322 patients	Watson for Oncology	Degree of agreement for treatment recommendations between WFO and 7-member multidisciplinary team
Yamazaki et al., 2020 [79]	Laparoscopic gastrectomy videos	10,716 images from 52 laparoscopic gastrectomy videos	Neural Network Platform, YOLOv3	Ability to detect and classify surgical instruments
Kitaguchi et al., 2019 [80]	Static images of laparoscopic gastrectomies	1242 captured images from 41 patients who underwent radical gastrectomy	Deep learning instance segmentation model (Mask R-CNN)	Intersection over union: how closely the AI predicted pancreas contour matches the true regions notated by surgeons
Takeuchi et al., 2023 [82]	Clinical data and surgical videos of patients who underwent robotic distal gastrectomy (RDG)	56 patients who underwent RDG with D1 or D2 lymphadenectomy	Multi-stage temporal convolutional network (TeCNO)	Performance of AI model to predict operation complexity in comparison with
Chien et al., 2008 [84]	Pre-and postoperative clinical data from patients with postgastrectomy complications	521 patients	Artificial Neural Network (ANN), Decision Tree (DT), Logistic Regression (LR)	Prediction of postoperative complications

## 4. Discussion: Strengths and Upcoming Challenges

In the current era of gastro-oncology, where advances in early detection and primary oncological treatment have enabled a shift towards organ-sparing approaches, the design of AI systems trained and validated for the accurate detection of recurrence or residual disease is of paramount importance [85,86]. To achieve this, extensive datasets comprising patient information, including clinical records, endoscopic images, and other imaging data, must be collected [87]. These datasets should cover a wide range of variables such as demographic factors (age, sex, ethnicity, and comorbidities), oncological factors (chemoradiotherapy regimens), surgical details (approach, technique, and extent of lymphadenectomy), and tumor-specific parameters (histology, preoperative and pathological TNM staging, and tumor grade) [87]. In addition, the AI system must undergo rigorous validation using independent external datasets to ensure reliability and generalizability.

The main strengths of the implementation of artificial intelligence are concluded in Table 4 and focus mainly on the diagnostic and screening ability of vast data sources with acceptable sensitivity. This establishes deep learning as a helpful tool at the forefront of population-based screening procedures, allowing for the handling of more complex and obscure cases than are possible with human evaluation. Especially in developing countries with limited resources, automated screening tools could essentially reduce the amount of missed cases by identifying key image features. In staging and prognosis, the strength of DL models relies on the assessment of histopathological phenotypes and molecular biomarkers with prognostic significance that can be used to stratify patients and suggest adequate personalized treatment plans.

However, technical limitations remain in studies using DL to detect early gastric cancer recurrence [88]. While incorporating larger numbers of consecutive video frames into training the AI programs improves comprehensive coverage of malignant gastric lesions, it also introduces “noise”, which can affect diagnostic accuracy and requires additional targeting and localization of the imaging background [89,90]. Moreover, when implementing AI in the endoscopic workflow, defining and setting clear and realistic objectives is essential, ensuring that the software is compatible with the endoscopy system hardware [91]. Despite the vast opportunities for automation, most current approaches rely on supervised learning, which depends on finely human-labeled data—experts annotate images by marking lesion areas, select the appropriate algorithms, and label datasets to train CNNs [92]. Consequently, doctors across different specialties and healthcare systems need to receive consistent training to use deep learning systems, interpret results, and apply feedback so that the models remain updated and accurate.

The wide variety of data formats and encoding methods across different healthcare facilities, systems, and devices further complicates data sharing and integration. This underscores the need for larger, multicenter, and cross-regional datasets to effectively assess AI performance [93]. However, data from different institutions frequently exhibit distribution shifts due to differences in imaging protocols, equipment, and patient populations. Such inconsistencies challenge model training, as machine learning generally assumes all data are drawn from the same distribution [94]. To address this, it is essential to establish and adopt standardized scanning protocols for gastric imaging. Given the sensitive nature of medical data, strict adherence to data protection regulations such as the General Data Protection Regulation (GDPR) and the Health Insurance Portability and Accountability Act (HIPAA) is also required to safeguard patient privacy [95].

Implementing such systems in clinical practice also demands the development of appropriate infrastructure, consideration of cost-effectiveness, and acceptance by key stakeholders, including both clinicians and patients, especially in Western populations where gastric cancer is less prevalent [96,97]. Τhese requirements highlight the critical need for large-scale, well-structured randomized clinical trials carried out through collaborative efforts to fully unlock the potential of AI in healthcare. Despite the fact that all of the 19 AI trials involving gastric cancer have been completed or are in progress, most of these AI models have yet to be adopted in real-world clinical settings, and their practical impact remains limited (Table 4) [98]. The publication of these studies has also highlighted challenges such as biased datasets, limited model interpretability, risks of false positives and negatives, and ethical issues related to patient privacy, regulatory approval, and clinician responsibility [99].

## 5. Conclusions

The aim of this study was to provide a clear and comprehensive overview of the current applications and strengths of emerging artificial intelligence (AI) technologies in the clinical evaluation of gastric cancer. This review enables readers to develop an integrated understanding of how large datasets can be organized, processed, and utilized as pipelines for the development of deep learning systems (Table 5). Key areas of application include early detection, pathological analysis, risk assessment, treatment planning, and outcome prediction. Although several challenges remain, integrating AI technologies into clinical practice holds significant promise. Achieving this goal will require strong multidisciplinary collaboration, as well as large-scale randomized controlled trials to validate the effectiveness and reliability of AI models.

## Figures and Tables

**Table 4:** Ongoing registered clinical trials on artificial intelligence in gastric cancer diagnosis and treatment.

Clinical Trial ID	Population	Intervention	Clinical Outcome	AI Software Used	Status
NCT06971471 (AIMING study)	>60 years-old patients undergoing upper gastrointestinal endoscopy for selected indications at areas with high-risk of gastric cancer	Integration of AI assistance in screening gastroscopy	Miss rate reduction: change in the miss rate of early gastric cancer and dysplastic lesions at upper endoscopy when using AI assistance	Core work packages (WPs): WP1, WP2, WP3, WP4	Not yet recruiting
NCT06275997 GAIN project	>60 years-old patients undergoing upper gastrointestinal endoscopy for selected indications at areas with high-risk of gastric cancer	Integration of AI assistance in screening gastroscopy	Miss rate reduction: change in the miss rate of early gastric cancer and dysplastic lesions with upper endoscopy when using AI assistance	Core work packages (WPs): WP1, WP2, WP3, WP4	Not yet recruiting
NCT05447221 2022-SDU-QILU-110	Patients aged 40–75 years old who undergo the gastroscopy examination and biopsy at Qilu Hospital, Shandong Univesity	Pathologists and AI will assess the severity of intestinal metaplasia with whole slide images of gastric biopsies	The diagnostic performance of the AI model to assess the severity of intestinal metaplasia in a single biopsy tissue slide	Digital Pathology artificial intelligence diagnosis systems (DPAIDS)	Currently recruiting
NCT06495645 Protocol_upper_RCTV10	Patients > 40 years old scheduled for elective upper endoscopy	AI-assisted upper gastrointestinal endoscopy	The diagnostic miss rate: the number of newly detected gastric neoplasia in the second examination divided by the total number of gastric neoplasia detected in both examinations for each patient	NA	Currently recruiting
NCT05819099 2023SDU-QILU-1	Patients aged 18 to 75 years old who underwent endoscopic examination or treatment with pathologically confirmed esophageal gastric junction adenocarcinoma at Qilu Hospital, Shandong University	Pathologists and AI will assess the severity of intestinal metaplasia with whole slide images of gastric biopsy	The diagnostic performance of the AI model when assessing the severity of intestinal metaplasia in a single biopsy tissue slide	Digital Pathology artificial intelligence diagnosis systems (DPAIDS)	Νοt yet recruiting
NCT05368636 2022SDU-QILU-G001	Patients aged 40 to 80 years old who are scheduled for gastroscopy	Observational study	Sensitivity and specificity of artificial intelligence models	NA	Not yet recruiting
NCT04675138 2022SDU-QILU-G001	Patients aged 21 years old and older with primary gastric adenocarcinoma or patients with gastroesophageal junction cancers or esophageal cancer	Development of an alternative Clinical Decision Support Systems (CDSS) for oncology therapy selection	NA	Concordance Rate: Comparative agreement in recommendations between the two study groups	Not yet recruiting
NCT04840056 2021.082	Patients aged 18 years old and older, with histologically proven atrophic gastritis or intestinal metaplasia (at antrum and/or body and/or angular of stomach)	Development of an alternative Clinical Decision Support Systems (CDSS) for oncology therapy selection	NA	Concordance Rate: Comparative agreement in recommendations between the two study groups	Not yet recruiting
NCT05916014 2022SDU-QILU-123	Patients aged 18 years old and older, who undergo the white light endoscope examination at Qilu Hospital, Shandong University	Endoscopists and AI will assess the Kimura–Takemoto classification independently	NA	Accuracy, sensitivity, or specificity	Recruiting
NCT06632886 AI-MCScreen	Patients aged 18 years old and older who have undergone an abdominal or chest non-contrast CT scan	AI-assisted Non-contrast CT for Multi-Cancer Screening	NA	Diagnostic yield, incidence, resectable rate	Recruiting
NCT05426135 Jin_cancer risk	Patients aged 18 years old to 75 years old with suspected lung/stomach or colorectal cancer/lesions	Observational study	NA	The outcome of clinical diagnosis of suspected patients with stomach cancer and lesions	Recruiting
NCT06506825 HBGCCN	Patients aged 18 years old and older with gastric cancer at the Fourth Hospital of Hebei Medical University	Retrospective observational study	Gastric cancer AI-driven data integration genomic analysis	5-year overall survival	Recruiting
NCT05722275 CASMI003	Patients aged 18 years old and older with diagnosis of advanced gastric cancer (>cT3) by endoscopy–biopsy pathology, with both enhanced CT and laparoscopy, without typical peritoneal metastasis	Extracting and combining the radiomics features related to peritoneal metastasis of gastric cancer	Radiomics for gastric cancer	AUC of the intelligent analysis system in predicting peritoneal metastasis for gastric cancer	Recruiting
NCT06078930 IRB-2021-289	Patients aged 18 years old until 90 years with histologically and cytologically confirmed gastric cancer with no prior oncological therapy	Extracting and combining the radiomics on tongue imaging, tongue coating, saliva, gastric juice, and feces	Radiomics for gastric cancer	The differences in tongue images, tongue coating, saliva, gastric juice, and fecal samples between patients with gastric cancer and healthy individuals	Recruiting
NCT03452774 SYNERGY-AI	All patients with hematological and solid malignancies from the SYNERGY	A proprietary application programming interface (API) linked to existing electronic health records (HR) is used for dynamic matching based on CT allocation and availability for optimized matching	VTB (virtual tumor boards) program	Proportion of patients eligible for clinical trial enrollment (CTE)	Recruiting
NCT06534814 FUTURE08	Patients aged 18 years and older with confirmed diagnosis of gastric cancer and lymph node involvement	Application of artificial intelligence system to enhance the identification and characterization of lymph node metastasis	AID-GLNM	Identification of metastatic perigastric lymph nodes before surgery	Recruiting
NCT06478368 FUTURE06	Patients aged 18 years and older with Locally Advanced Gastric Cancer (LAGC) with consent to provide intraoperative dynamic video and a scheduled surgical treatment	Application of artificial intelligence system to enhance the identification and characterization of lymph node metastasis	NA	Peritoneal metastasis	Not yet recruiting
NCT05762991 202111108RINC	Patients aged between 20 years old and 80 years old with scheduled urea breath test and endoscopy	Application of artificial intelligence system to analyze the correlation between endoscopic images and urea breath test/histopathological tests	NA	Sensitivity to detect premalignant gastric lesions	Recruiting
NCT05762991 202111108RINC	Patients aged between 20 years old and 80 years old with scheduled urea breath test and endoscopy	Application of artificial intelligence system to analyze the correlation between endoscopic images and urea breath test/histopathological tests	NA	Sensitivity to detect premalignant gastric lesions	Not yet recruiting

**Table 5:** Summary of the main uses, strengths, and limitations of artificial intelligence models in gastric malignancies.

Summary of the Main Uses, Strengths, and Limitations of Artificial Intelligence Models in Gastric Cancer			
Clinical Uses	Key Metrics	Strengths	Limitations
Endoscopy	Identify and segment pathological sites, classification of precancerous changes, and tumor invasion depth for early gastric cancer	Higher sensitivity and specificity than endoscopists in internal validation sets	False positive results in AI models highly trained to recognize malignancy; still images with no real-time navigation; limited performance in external validation sets and for highly inflamed compartments
Radiology	Lymph node involvement assessment, metastasis detection, and intratumoral heterogeneity	Strong discriminatory performance, high predictive power, image-based performance, and no need for clinical characteristics	Vulnerable in different biological and histopathological tumor characteristics; need for international imaging archive
Pathology	Histopathological classification, tumor microenvironment (TME), immunophenotype, and molecular classification (EBV and HER-2 status)	High diagnostic accuracy and high concordance rate with pathologists and prognostic algorithms	Long processing time and costly tissue elaboration; limited performance in tumors with a high combined positive score (CPS); performance deviations depending on patients’ clinical characteristics; high misclassification rates in poorly differentiated tumor issue and stromal fibrosis
Prognosis	Cox proportional hazard models for calculation of outcomes and treatment response	Complex multivariate algorithms and high concordance rate with clinical pathologists	High discrepancy rate with outliers such as extremely older patients and stage IV disease; limited generalizability among countries and heath care systems
Surgery	Detect and replicate surgical instruments, predict critical momentum during surgery, identify surgical contour of anatomical regions, and prediction of postoperative complications	Effective at capturing the complex, nonlinear relationships between clinical variables and postoperative outcomes and high concordance rates between experienced surgeons	Limited available real-time processing studies; limited data on performance in external validation sets

## Data Availability

No new data were created or analyzed in this study. Data sharing is not applicable to this article.
